# Rapid, Strong, and Visible Efficacy of a Dermocosmetic in Acne Patients With Dark Skin Phototypes: Results of a Randomized Split‐Face Study

**DOI:** 10.1111/jocd.70737

**Published:** 2026-03-02

**Authors:** Catherine Queille‐Roussel, James Odeimi, Margot Broallier, Delphine Kerob, Jerry Tan

**Affiliations:** ^1^ CPCAD, CHU L'archet Nice France; ^2^ Laboratoire Dermatologique La Roche‐Posay France; ^3^ Department of Medicine and Windsor Clinical Research Inc Western University Windsor Canada

**Keywords:** acne, dark skin phototype, dermocosmetic, kinetics

## Abstract

**Introduction and Objectives:**

Acne is a chronic inflammatory skin disease that can cause acne‐induced hyperpigmentation (AIH), especially in subjects with phototype IV and above (hereafter darker skin tones).

This study evaluated the efficcay kinetics of a dermocosmetic cream (DC cream) in adults with darker skin phototypes and mild to moderate acne.

**Material and Methods:**

An intra‐individual, randomized, split face, single centre 57 day‐study was conducted in 16 adults with dark skin tones (phototype IV, V and VI) and mild‐to‐moderate acne. DC cream was applied 2/day on one hemiface. Assessments included total, inflammatory and non‐inflammatory lesion counts, AIH intensity and darkness severity, PAHPI score and local tolerance. Subjects rated the perceived benefits of DC cream.

**Results:**

75% of subjects were women, mean age was 26.8 ± 6.7 years; 50% had phototype IV, 31.2% phototype V, and 18.8% phototype VI. 68.8% had mild (GEA 2) and 31.2% moderate (GEA 3) acne.

The total lesion count significantly decreased from Day 5 (−17.1%; *p* < 0.05) until Day 57 (−44.9%; *p* < 0.01) on the DC‐treated side *versus* *−* 28.4% on the untreated side at Day 57. Inflammatory and non‐inflammatory lesion counts significantly (*p* < 0.01) decreased from Day 11 to Day 57 with DC cream. AIH marks intensity significantly (*p* < 0.01) decreased starting Day 11 until Day 57; as did the PAHPI score (−22.7%) after 57 days with hemi‐face differences being significantly (*p* < 0.05) in favor of DC cream. AIH darkness severity significantly improved with DC cream, with no changes on the untreated side. DC cream was highly appreciated and very well tolerated by the subjects.

**Conclusions:**

This clinical study provides strong evidence on the efficacy kinetics of a DC cream in acne management in subjects with dark skin tones. It shows that early, daily and specific treatment with a targeted DC significantly improves all acne lesions type, as well as AIH marks as soon as 11 days of use, in addition to exhibiting high patient satisfaction rates and excellent tolerability.

## 1 Introduction

*Acne vulgaris* and its sequelae are consistently reported among the most common dermatologic diagnoses for which people with skin of color (SOC) consult a dermatologist [1, 2]. While its etiology is similar across all phototypes, a key difference lies in its severity and prevalence, as well as the high risk of sequelae in darker phototypes, particularly acne‐induced hyperpigmentation (AIH) [3]. AIH occurs in 45.5% to 87.2% of subjects with darker phototypes (IV‐VI), and often presents as a greater concern than the acne itself [4]. Because AIH can persist for months or even years, it may progress to permanent scarring if the underlying inflammation persists or remains unresolved, leading to significant adverse psychosocial outcomes and severely impacting patient quality of life [2, 5].

Despite a plethora of data on general acne development and updated international guidelines on acne management, limited information exists regarding the day‐by‐day evolution of acne lesions lifecycle when managed with a dermocosmetic [6]. Furthermore, although topical treatments and dermocosmetics are widely recommended in patients with mild to moderate acne, there is a paucity of studies specifically investigating dermocosmetic use for acne in darker skin tones (Phototypes IV–VI) [2, 6, 7].

A recent 15‐day split‐face study investigated the efficacy kinetics of a dermocosmetic cream (Effaclar Duo+, La Roche‐Posay Laboratoire Dermatologique, France, hereafter DC cream) in fair‐skinned (Phototypes II/III) subjects with mild to moderate acne compared to no treatment for 57 days [8]. The DC cream contains multi‐targeted active ingredients— *Punica granatum* Pericarp extract, Salicylic acid, Niacinamide, Zinc gluconate, and *Aqua Posae Filiformis*. All ingredients have individually shown their benefit as monotherapy in improving acne lesions as well as AIH [9, 10, 11, 12, 13].

However, that prior research did not assess the kinetics of acne lesions progression in response to a dermocosmetic cream in darker skin phototypes, where mild to moderate acne and AIH present distinct challenges regarding acne treatment‐induced irritation, acne presentation and its clinical management, demanding dedicated and thorough investigation [2].

The present study assessed the kinetics of evolution of facial acne lesions and AIH marks in subjects with darker skin tones and mild to moderate acne treated with dermocosmetic cream compared to no treatment for 57 days.

## 2 Material and Methods

This single‐centre, intra‐individual, randomized exploratory study did not require approval from an ethics committee. Nevertheless, the study complied with all legal requirements for the conduct of clinical studies and subjects provided written informed consent prior to participation.

Adult subjects above 18 years of age with dark skin tones and mild to moderate acne according to the GEA (Global Acne Evaluation) grading system were to be recruited [14]. For each eligible subject, the hemiface to be treated (right or left) was determined according to a randomization list. Hemifaces of suitable subjects were randomized to either DC or remained untreated for 57 days. Subjects were asked to apply DC twice daily, in the morning and evening, to the to‐be‐treated hemiface. Prior to application of DC cream in the morning, subjects were to wash their entire face with a neutral cleanser (Toleriane Purifying Foaming Cleanser, La Roche‐Posay Laboratoire Dermatologique, France). During the first 2 weeks (from Day 1 to Day 13), product applications were performed at the study center once a day (except on Sundays) to ensure compliance. Subjects were advised to apply a SPF30 sunscreen on the face every morning at least 15 min after having applied DC cream; applications could be repeated during the day in case of sun exposure. All assessments were performed prior to the application of DC cream.

Evaluation visits took place every day from Day 1 to Day 6 then twice a week for 3 weeks (Day 8, Day 11, Day 15, Day 18, Day 22, and Day 25) and finally once a week up to Day 57. They included total, inflammatory, non‐inflammatory acne lesions count AIH marks count, AIH intensity and darkness severity as well as PAHPI (post‐acne hyperpigmentation index) scoring on both hemifaces [15]. Local tolerance to the DC was also assessed by the investigator (desquamation, dryness, and erythema) and the subjects (sensations of burning, itching, and tingling) on a scale from none to severe. The subjects also rated the perceived benefits of the DC cream using a questionnaire at Day 57.

Standardized, full face and both profiles images using Colorface (Newtone, France) based on multimodal acquisition as cross‐polarization mode were taken at pre‐defined visits during the study.

Categorical data were summarized using the number and percentage of subjects in each category. Continuous data were summarized using the arithmetic mean, SD, CV (%), minimum, median, and maximum values. Quantitative parameters were analyzed using a mixed‐effect model. This model included time point, treatment, the interaction time point*treatment, and baseline as fixed effects and subject added as a random effect. The comparisons between post‐baseline time point and baseline for each treatment were performed using a Dunnett adjustment. The change from baseline was also analyzed using a mixed‐effect model. The comparison between treatments at each time point was performed whatever the interaction results using a Tukey adjustment. The analysis of the questionnaire was descriptive only. The probability level was set at 5%.

All statistical analyses were performed using R Software version 4.0.2.

## 3 Results

### 3.1 Demographic and Baseline Data

Sixteen subjects (12 females and 4 males) with phototype IV (8; 50.0%), V (5; 31.2%), or VI (3; 18.8%) were included and hemifaces were randomized. The mean age was 26.8 ± 6.7 years; 68.8% (11 subjects) had mild acne (GEA 2), and the remaining had moderate (GEA 3) acne. Detailed acne and skin quality data at study start are provided in Table 1.

**TABLE 1:** Day 1 acne and skin quality data.

		DC‐cream treated hemiface	Untreated hemiface
Total lesions	Mean ± SD	30.4 ± 11.5	29.00 ± 11.30
	Median	26.5	25
	Min; Max	17.0, 66.0	19.0, 67.0
Inflammatory lesions	Mean ± SD	10.4 ± 3.6	12.8 ± 3.1
	Median	10.5	13
	Min; Max	4.0, 17.0	7.0, 18.0
Non‐inflammatory lesions	Mean ± SD	17.6 ± 10.1	15.94 ± 10.1
	Median	15.5	13
	Min; Max	10.0, 54.0	9.0, 50.0
AIH count	Mean ± SD	8.7 ± 6.0	8.5 ± 5.2
	Median	7	5.5
	Min; Max	3.0, 24.0	3.0, 17.0
AIH intensity	Mean ± SD	3	3.3 ± 0.5
	Median	3.0, 3.3	3
	Min; Max	3.0, 4.0	3.0, 4.0
PAHPI score	Mean ± SD	11.0 ± 1.9	11.1 ± 1.8
	Median	11	11
	Min; Max	9.0, 15.0	8.0, 14.0

Abbreviations: AIH, acne‐induced hyperpigmentation; PAHPI, post‐acne hyperpigmentation index.

No statistically significant and no clinically meaningful difference between hemifaces were observed for any of the lesion counts at baseline/Day 1.

All hemifaces were comparable for GEA, AIH marks, and PAHPI.

### 3.2 Clinical Parameters

**FIGURE 1:**
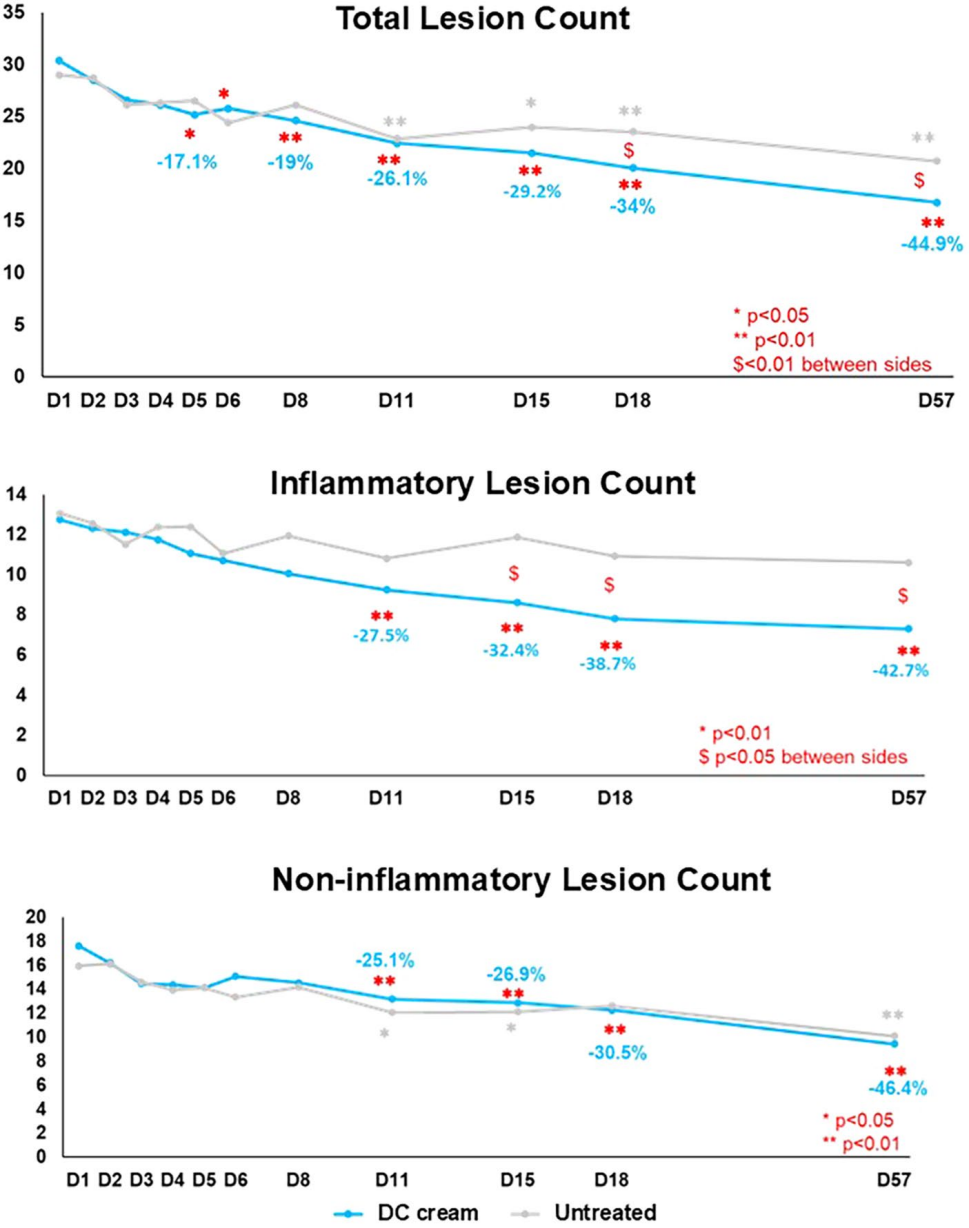
Acne Lesion Counts evolution over time from Day 1.

Figure 1 shows the evolution of the total, inflammatory and non‐inflammatory lesions count from Day 1 to Day 57.

At Day 57, differences between hemifaces were superior to at least one lesion for all lesion types mean counts (total lesion count: DC cream: 16.8 ± 11.3, untreated: 20.8 ± 8.1, inflammatory lesion count: DC cream: 7.3 ± 3.7, untreated: 10.6 ± 3.1, non‐inflammatory lesion count: 7.9 ± 5.5, untreated: 8.8 ± 5.5); all differences were statistically significant (*p* < 0.001) and clinically relevant for the total and inflammatory lesions count.

Total lesions count had significantly decreased with DC cream as soon as Day 5 (−17.1%, *p* < 0.05) and overtime until Day 57 (−44.9%, *p* < 0.001); lesion improvement on the untreated side started later at Day 11. The between sides comparison showed a statistically significant (*p* = 0.006) higher improvement with DC at Day 18 and Day 57.

Inflammatory lesion count showed statistically significant reduction with DC cream compared to Day 1/baseline, at Day 11 (−27.5%, *p* = 0.005), Day 15 (−32.4%, *p* < 0.001), Day 18 (−38.7%, *p* < 0.001), and Day 57 (−42.7%, *p* < 0.001) while on the untreated side, no significant improvements were observed. Hemifaces comparisons showed that changes were significant in favor of DC cream from Day 15 till Day 57 (*p* < 0.001).

Non‐inflammatory lesion count on the DC‐treated hemiface showed a statistically significant decrease at Day 11 (−25.1%, *p* = 0.04), Day 15 (−26.9%, *p* = 0.001), Day 18 (−30.5%, *p* < 0.001), and Day 57 (−46.4%, *p* < 0.001). On the untreated side, a significant decrease was observed at Day 11 (−24.3%, *p* = 0.019), Day 15 (−24%, *p* = 0.023), and Day 22 (−30.9%, *p* = 0.002). Starting Day 11 and lasting until Day 57, the decrease from Day 1/baseline was significant (*p* = 0.001). No statistically significant changes between hemifaces were observed at any visit timepoint.

Regarding AIH, no significant change in the AIH lesion count was observed on either hemiface throughout the study. However, a significant decrease in AIH intensity was noted with the DC cream treatment as early as Day 11 (−19.4%, *p* < 0.01), progressing incrementally until Day 57 (−38.5%, *p* < 0.01) whereas no significant decrease was observed on the untreated side at any time during the study. Significant differences in AIH intensity between the treated and untreated hemifaces were observed at Day 11, Day 15 (*p* = 0.049), Day 18 (*p* = 0.003), Day 22 (*p* = 0.006), and again from Day 36 to Day 57 (all *p* ≤ 0.05). Furthermore, according to investigator assessments at Day 57, AIH darkness severity was significantly improved in 100% of subjects on the DC cream‐treated hemiface, whereas no improvement whatsoever was observed on the untreated hemiface.

**FIGURE 2:**
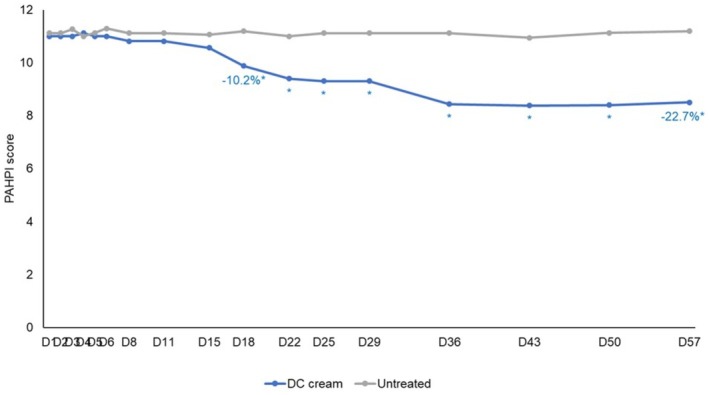
Evolution of the PAHPI score on the DC‐cream treated and untreated hemiface over time.

PAHPI score showed a significant (*p* < 0.01) decrease compared to baseline starting Day 18 (−10.2%) until Day 57 (−22.7%) on the DC treated hemiface with no change on the untreated hemiface (Figure 2). The between‐hemiface differences of the PAHPI score were significant (*p* < 0.001) in favor of DC cream starting Day 18 and lasting until Day 57.

According to the investigator, DC cream was very well tolerated with 88% of subjects experiencing no desquamation and more than 77% experiencing no erythema nor skin dryness. According to the subjects, 88% experienced no itching and 83% reported no burning sensations following DC cream use for 57 days.

**FIGURE 3:**
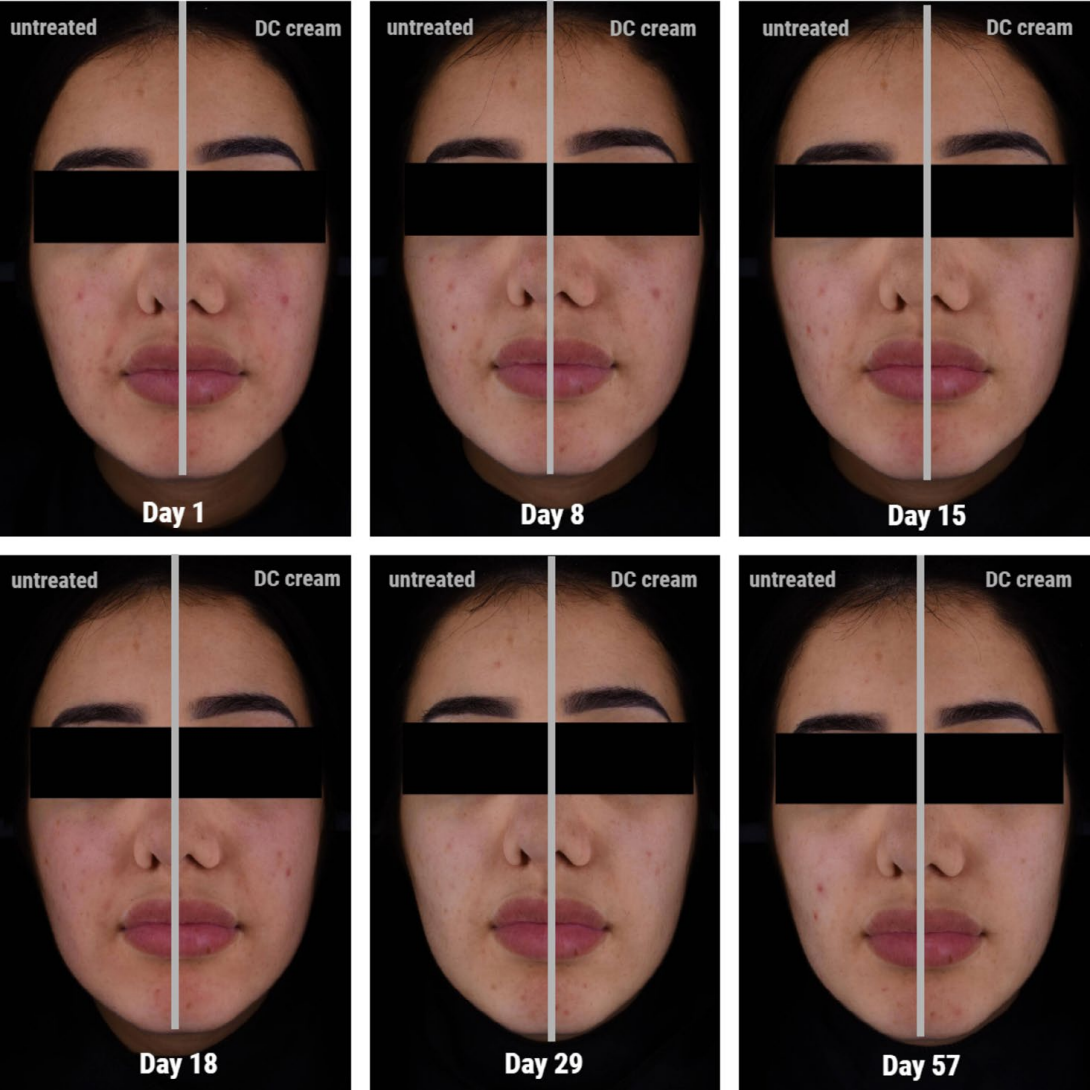
Kinetics of changes of lesions and marks over time on DC‐treated side (Phototype IV) analyzed using Colorface (average case). Left hemiface: DC cream‐treated, right hemiface: untreated. At baseline/Day 1, this subject had 17 lesions on the treated and 23 on the untreated side. After 57 days, lesion counts decreased to 6 on the treated side and 14 on the untreated side. The inflammatory lesions count decreased from 7 from baseline/Day 1 to 4 at Day 57 with DC cream and increased from 8 lesions to 10 on the untreated side over the same period of time.

An example (Phototype IV) of the evolution over time of acne lesions and AIH with the DC cream is given in Figure 3.

### 3.3 Subject Assessments

The DC cream was highly appreciated for its cosmetic attributes and benefits. All subjects (100%) confirmed it did not leave the skin greasy nor oily and was easy to apply. Additionally, 94% reported improved skin comfort, softness, and appearance and 88% observed a decrease in both the visibility and number of imperfections.

## 4 Discussion

This exploratory, intra‐individual, randomized study evaluated the kinetics of efficacy of a multi‐targeted DC cream in acne lesions and AIH marks evolution in adults with dark skin tones (IV to VI) presenting mild to moderate facial acne.

DC cream significantly improved all acne lesions and AIH intensity as soon as 11 days of use, demonstrating a rapid onset of action and sustained efficacy over 57 days of twice daily application. Moreover, results corroborate the understanding that dermocosmetics are beneficial in the management of mild to moderate acne, as they can foster improved treatment compliance. This, in turn, establishes their place within the therapeutic armamentarium for mild to moderate acne, irrespective of the subject's phototype [6].

While this study could not demonstrate a significant improvement of AIH lesion count overtime, DC cream allowed for a significant (*p* < 0.01) improvement of 38.5% of its intensity after 57 days. This result underlines the benefit of DC cream in the long‐term management of AIH. AIH is a common sequelae in acne patients with darker skin tones. It can heavily impact quality of life and contribute to high stigma [16, 17, 18]. Consequently, there is a crucial need for products that not only improve acne lesions but also effectively and rapidly address AIH intensity and visibility. Such DC treatments must simultaneously manage skin sensitivity in this demographic to prevent inflammatory marks from evolving into permanent scars.

Previous studies assessing dermocosmetic benefits have predominantly included fair skin populations or broad Fitzpatrick phototype cohorts [7, 19]. Another study assessed a DC routine equally in any skin phototype without assessing the post‐acne marks. In contrast, the present investigation exclusively focused on subjects with darker skin phototypes, acknowledging their specific clinical considerations. This focus is critical because acne in individuals with darker skin tones has historically received limited research attention, despite the universal prevalence of acne across all ages and phototypes [20]. The results presented demonstrate that the DC cream effectively and safely improves both acne and AIH without altering natural baseline skin tone—a critical advantage given the inherent risks of hypopigmentation or irritation associated with depigmenting or exfoliating agents often used to manage AIH in skin of color [4].

Consequently, the DC formula demonstrated rapid, strong and visible efficacy in improving all acne lesions and AIH intensity. The DC cream was very well tolerated among individuals with darker skin phototypes and was highly appreciated for its perceived benefits; collectively, these attributes hold the potential to significantly enhance treatment adherence, a critical factor in the long‐term management of acne and subsequent patient outcomes [21].

While this exploratory study yielded positive results, its primary limitation is the small sample size, which restricts the generalizability of our findings. Given these encouraging preliminary data, a larger, multicentric study focusing on adult subjects with dark skin phototypes is warranted to further explore this significantly under‐investigated area.

In conclusion, this exploratory split‐face study provides insights into the kinetics of acne lesions and AIH in darker skin phototypes in response to a specific DC cream use. It effectively demonstrates that early and adapted management as well as education and simplified treatment regimens lead to significant, visible, and rapid improvement in both acne lesions and marks over time.

## Author Contributions

M.B., J.O., and D.K. designed the study and supervised its conduct. C.Q.‐R. conducted the study. J.O. wrote the manuscript and coordinated the different writting activities with the authors and the journal. All authors analysed the data, provided inputs, read and approved the manuscript.

## Funding

This study was sponsored by La Roche‐Posay Laboratoire Dermatologique, France.

## Ethics Statement

This non‐interventional, single centre, intra‐individual and exploratory study did not require approval from an ethics committee. However, the study complied with all legal requirements for the conduct of clinical studies and subjects provided written informed consent prior to participation.

## Conflicts of Interest

All authors apart from C.Q.‐R. and J.T. are employees of La Roche‐Posay Laboratoire Dermatologique. C.Q.‐R. has no conflicts of interest to disclose. J.T. is a consultant and speaker for La Roche Posay, Laboratoire Dermatologique, France.

## Data Availability

The data that support the findings of this work are available from the corresponding author upon reasonable request.
